# Cardiac Rehabilitation in Peripheral Artery Disease in a Tertiary Center—Impact on Arterial Stiffness and Functional Status after 6 Months

**DOI:** 10.3390/life12040601

**Published:** 2022-04-18

**Authors:** Razvan Anghel, Cristina Andreea Adam, Dragos Traian Marius Marcu, Ovidiu Mitu, Mihai Roca, Grigore Tinica, Florin Mitu

**Affiliations:** 1 Clinical Rehabilitation Hospital, Cardiovascular Rehabilitation Clinic, Pantelimon Halipa Street nr 14, 700661 Iași, Romania; razvan0312@gmail.com (R.A.); adam.cristina93@gmail.com (C.A.A.); roca2m@yahoo.com (M.R.); mitu.florin@yahoo.com (F.M.); 2 Department of Internal Medicine, University of Medicine and Pharmacy “Grigore T. Popa”, University Street nr 16, 700115 Iași, Romania; dragos.marcu11@yahoo.com; 3 “Sf. Spiridon” Clinical Emergency Hospital, Independence Boulevard nr 1, 700111 Iași, Romania; 4 Department of Cardiovascular Surgery, University of Medicine and Pharmacy “Grigore T. Popa”, University Street nr 16, 700115 Iași, Romania; grigoretinica@yahoo.com; 5 Institute of Cardiovascular Diseases “Prof. Dr. George I.M. Georgescu”, 700503 Iași, Romania

**Keywords:** peripheral artery disease, cardiac rehabilitation, arterial stiffness, quality of life, adherence, exercise, ambulatory arterial stiffness index

## Abstract

*Background and Objectives:* Cardiac rehabilitation (CR) plays an essential role in peripheral artery disease (PAD), leading to improved functional status, increased quality of life, and reduced arterial stiffness. We aimed to assess factors associated with clinical improvement 6 months after enrolment in a rehabilitation program at an academic medical center in north-eastern Europe. *Materials and Methods*: We conducted a prospective cohort study on 97 patients with PAD admitted to a single tertiary referral center. At the 6-months follow-up, 75 patients (77.3%) showed improved clinical status. We analyzed demographics and clinical and paraclinical parameters in order to explore factors associated with a favorable outcome. *Results*: Hypertension (*p* = 0.002), diabetes mellitus (*p* = 0.002), dyslipidemia (*p* = 0.045), and obesity (*p* = 0.564) were associated with no clinical improvement. Smoking cessation (*p* < 0.001), changing sedentary lifestyle (*p* = 0.032), and improvement of lipid and carbohydrate profile as well as functional status parameters and ambulatory arterial stiffness index (*p* = 0.008) were factors associated with clinical improvement at the 6-months follow-up. *Conclusions*: PAD patients require an integrative, multidisciplinary management to maintain functional status and increase quality of life. Improving carbohydrate and lipid profile, adopting a healthy lifestyle, quitting smoking and increasing exercise capacity are predictors for clinical improvement 6 months after enrolment in a CR program.

## 1. Introduction

Peripheral artery disease (PAD) has a high prevalence among cardiovascular diseases, but is often underdiagnosed or undertreated, which occurs secondary to atherosclerotic damage to peripheral arteries [1]. Despite therapeutic advances (both interventional and medical) it remains a public health issue due to the large number of patients affected, the unfavorable prognosis, and the costs associated with the treatment of complications or cardiac rehabilitation (CR) programs [2].

Cardiovascular diseases (CVD) remain the main cause of mortality and morbidity in industrialized countries despite notable technological advances [3]. The management of PAD patients is complex and requires an integrative, multidisciplinary, and personalized approach for each patient. The correction of modifiable cardiovascular risk factors, adherence to drug treatment, surgical or interventional revascularization of stenotic lesions, or enrollment in specialized CR programs are premises for a favorable prognosis [2]. Physical exercise is an essential element of CR that contributes to maintaining functional status and prevents functional decline. CR programs should be seen as a “therapeutic puzzle”, in which dietary advice, smoking cessation programs, weight management, lipid and carbohydrate profile control, and blood pressure management contribute to improving both physical fitness and quality of life [4,5].

Arterial stiffness is a predictor of cardiovascular mortality and morbidity. It is widely used nowadays used to assess target organ damage in hypertensive patients [6,7]. Both arteriosclerosis and atherosclerosis are strongly associated with arterial stiffness, with a known relationship between it and CVD mortality [8,9]. Several methods are known for measuring arterial stiffness, the most common being measuring the pulse-wave at two points of known distance (common carotid and femoral arteries) to obtain the pulse-wave velocity (PWV) [10]. PWV is considered to be the gold-standard technique in assessing arterial stiffness, but the ambulatory arterial stiffness index (AASI) is considered to be a useful, non-invasive method. Based on the fact that both systolic and diastolic pressures increase simultaneously in a compliant artery, while for a stiff artery the increase in systolic pressure is accompanied by a lower increase or even decrease of diastolic pressure, measuring the regression slope of diastolic-on-systolic blood pressure measurements in a 24-h ambulatory continuous monitoring has been considered an indirect marker of arterial compliance [9,11].

Lifestyle changes, proper diet, and drug administration as well as awareness regarding the natural evolution of the disease are essential components of any CR program. The aim of this study was to assess the clinical and paraclinical factors associated with improvement in functional status 6 months after the start of the CR program.

## 2. Materials and Methods

### 2.1. Study Design

**Figure 1:**
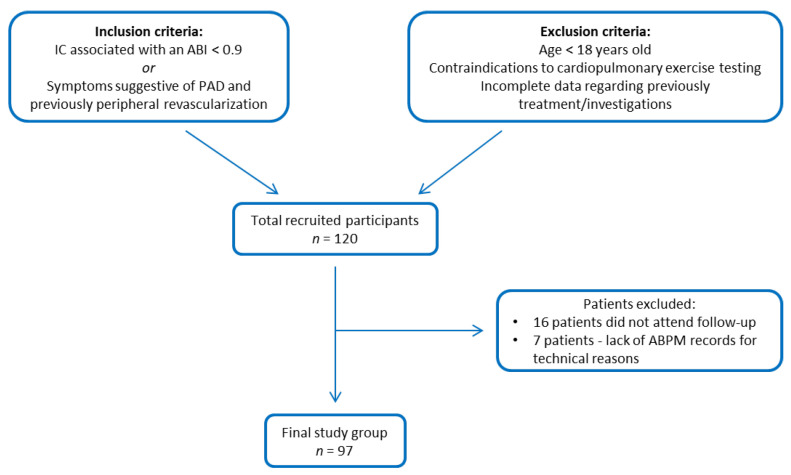
Flow chart of the studied group (IC: intermittent claudication; ABI: ankle-brachial index; PAD: peripheral artery disease; ABPM: ambulatory blood pressure monitoring).

We conducted a prospective cohort study on 120 patients admitted to a single tertiary referral center in North-East Romania, with a diagnosis of PAD. The diagnosis was based on the presence of intermittent claudication (IC) and an ankle-brachial index (ABI) less than 0.9 or in the presence of symptoms suggestive of PAD and previous history of peripheral revascularization. The exclusion criteria were: age less than 18 years old, patients with contraindications for cardiopulmonary exercise testing, or those with incomplete data regarding treatment or assessed investigations. Of the 120 patients initially enrolled, 16 were excluded due to follow-up drop-out, and for seven patients, due to technical reasons, it was not possible to perform 24 h ambulatory blood pressure monitoring at the 6-month follow-up. The final study group comprised 97 patients (Figure 1).

Patients underwent an extensive evaluation prior to enrolment in the study, which included laboratory tests, cardiopulmonary exercise testing, and ambulatory blood pressure monitoring for 24 h. For the 97 PAD patients enrolled, demographics, personal medical history, tobacco and alcohol consumption habits, and chronic medication were obtained from the observation charts. We used the current guidelines to evaluate and define comorbidities such as arterial hypertension [12], heart failure [13], chronic kidney disease [14,15], and diabetes [16]. The laboratory tests included lipid profile, serum glucose, C reactive protein (CRP), and glycated hemoglobin (HbA1C), the results being presented according to the International System of Units. Body mass index (BMI) was calculated as the ratio of weight (kg) and height (m^2^). AASI was defined as 1 minus the regression slope of diastolic on systolic blood pressure (BP) values derived from a 24-h ambulatory blood pressure monitoring (ABPM) recording [17]. All patients underwent a cardiopulmonary exercise test to determine functional capacity and to assess recommended parameters for aerobic physical training.

At the 6-month follow-up biological samples, cardiopulmonary exercise testing and blood pressure monitoring were repeated. The 97 patients were divided into two groups on the 6-months follow-up, based on the presence or absence of clinical improvement assessed as staging in a class lower than at enrolment using the Fontaine classification system [18]. During the CR program, all patients received dietary advice, psychotherapy, and drug therapy (statins for dyslipidemia, hypoglycemic treatment for diabetic patients).

### 2.2. Statistical Analysis

Data were reported as the mean ± standard deviation (SD) and as a number (frequency or percentages). Continuous variables were compared using the *t*-test (parametric analysis). Categorical variables were compared using the Fisher Exact test. A *p*-value of ≤0.05 was considered statistically significant. To explore factors associated with clinical improvement at 6-month follow-up, a regression analysis was performed. Receiver-operator characteristic analyses were performed to calculate area under the curve for clinical parameters. The descriptive analysis was performed using SPSS statistics software (version 20 for Windows; SPSS Inc., Chicago, IL, USA).

### 2.3. Ethics

The study was approved by the Ethics Committee of the University of Medicine and Pharmacy “Grigore T. Popa” Iași and of the Clinical Rehabilitation Hospital Iași, and was conducted according to the Helsinki Declaration. All patients signed an informed consent statement which mentioned that the results would be used for research purposes.

## 3. Results

We analyzed 97 patients with PAD on admission and at the 6-month follow-up after the start of the CR program. According to the presence or absence of clinical improvement, we divided the 97 patients into two groups: those with a favorable evolution of the clinical status (with clinical improvement, 75 patients) and those without clinical improvement after the CR program (22 patients). The demographics and resting hemodynamics are presented in Table 1. Of the patients enrolled, 67% were males with a mean age of 69.66 ± 8.60 years old. Regarding anthropometric parameters, the average BMI at enrolment was 27.74 ± 3.65 kg/m^2^ with a decreasing value at the 6-month follow-up of 24.14 ± 3.17 kg/m^2^ (*p* = 0.037). Percentage of sedentary patients (71.1% vs. 52.6%, *p* = 0.032), smokers (64.9% vs. 29.9%, *p* < 0.001), and alcohol consumers (16.5% vs. 9.3%, *p* = 0.973) decreased after enrolment in the study. The 6-month assessment showed a decrease in systolic blood pressure values by 4.3% (140.79 ± 21.56 vs. 134.73 ± 20.63 mmHg, *p* = 0.131) and diastolic by 3.6% (80.15 ± 14.97 vs. 77.26 ± 14.43, *p* = 0.485), but without statistical significance for the analyzed group.

Comorbidities, symptoms, biological parameters and those associated with cardiopulmonary exercise testing were analyzed in association to clinical improvement. Hypertension (90.9% vs. 54.7%, *p* = 0.002), diabetes mellitus (86.4% vs. 49.3%, *p* = 0.002), dyslipidemia (77.3% vs. 53.3%, *p* = 0.045) and obesity (68.2% vs. 61.3%, *p* = 0.564) were more frequent in the group of patients without clinical improvement at the 6-month follow-up (Table 2). Patients in the first group also had pulmonary or vascular comorbidities such as coronary artery disease (59.1% vs. 46.7%, *p =* 0.310), chronic obstructive pulmonary disease (27.3% vs. 10.7%, *p* = 0.052) or venous insufficiency (77.3% vs. 57.3%, *p* = 0.092). Regarding clinical parameters, the improvement or disappearance of paresthesia (*p* = 0.005) and the feeling of cold feet (*p* = 0.001) and cold skin (*p* = 0.028) were parameters associated with favorable clinical outcomes after a 6-month CR program. Quitting smoking (68.2% vs. 81.3%, *p* < 0.001) and embracing a healthy lifestyle (72.7% vs. 46.7%, *p* = 0.032) improves the functional status of PAD patients.

**Figure 2:**
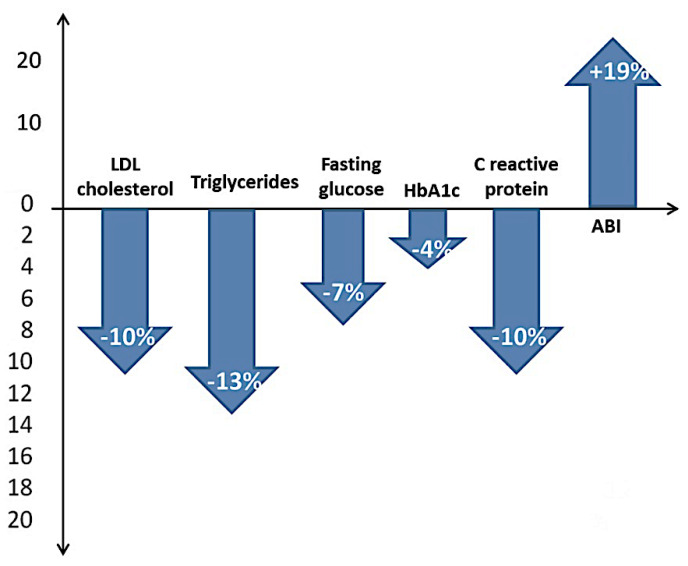
Parameters associated with clinical improvement at the 6-month follow-up. (HbA1C: glycated hemoglobin; LDL-cholesterol: low-density lipoprotein cholesterol, ABI: ankle-brachial index).

Table 3 shows blood biochemistry, exercise stress test results, and the blood pressure monitoring findings. The mean value of the serum level of total cholesterol decreased by 17% compared with the values at enrollment, with a more pronounced effect in patients with clinical improvement (*p* = 0.153). Statistically significant results were obtained for low-density lipoprotein (LDL) cholesterol (*p* = 0.004, decrease of 10%) and triglycerides (*p* = 0.005, decrease of 13%), as well as fasting glucose (*p* < 0.001, decrease of 7%) and HbA1C (*p* < 0.001, decrease of 4%) (Figure 2).

Cardiopulmonary exercise testing results after 6 months of aerobic physical training showed additional improvement among patients with clinical benefit. Thus, peak oxygen uptake (VO_2peak_) (14.95 ± 5.74 vs. 12.13 ± 3.94, *p* = 0.034), peak systolic BP (154.94 ± 18.11 vs. 164.76 ± 23.55, *p* = 0.040), and perception of effort on the Borg scale (13.12 ± 1.02 vs. 13.95 ± 1.29, *p* = 0.002) are statistically significant parameters associated with clinical improvement. At enrolment, patients were classified on the basis of cardiopulmonary exercise test results predominantly in Weber class C (48 cases, 49.5%), with predominantly moderate (31 cases, 32.0%) or moderately-severe (28 cases, 28.9%) functional limitation. At the 6-month follow-up, after regular physical training, the degree of functional limitation decreased, remaining predominantly moderate (29 patients, 29.9%), specific to Weber B class (42 cases, 43.3%). ABI values (0.84 ± 0.25 vs. 0.71 ± 0.24, *p* = 0.031), echocardiographic parameters, especially left ventricular ejection fraction (LVEF, %) (50.93 ± 9.93 vs. 44.03 ± 10.55, *p* = 0.006), and associated ABPM parameters improved, especially those correlated with arterial stiffness: pulse pressure (58.65 ± 11.54 mmHg vs. 51.62 ± 12.19 mmHg, *p* = 0.024) and AASI (0.42 ± 0.19 vs. 0.47 ± 0.11, *p* = 0.008).

**Figure 3:**
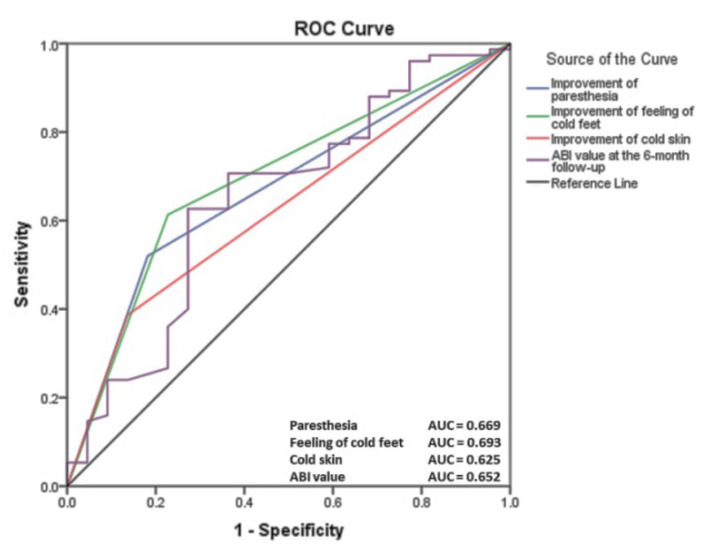
The receiver operating characteristic curve of the clinical signs and ABI value associated with clinical improvement (ABI: ankle-brachial index).

We identified a variety of clinical and paraclinical parameters associated with clinical improvement at the 6-month follow-up, which are presented in Table 4. Lowering fasting glucose by at least 7% (*p* = 0.001), HbA1C by at least 4% (*p* = 0.001), and lipid profile parameters such as LDL-cholesterol (*p* = 0.013) and triglycerides (*p* = 0.026) are associated with improved functional status. Physical training led to improvement in clinical signs such as paresthesia (*p* = 0.008) and feeling of cold feet (*p* = 0.003) or cold skin (*p* = 0.037). The receiver operating characteristic curve of the clinical signs and ABI values associated with clinical improvement identified positive predictive values of paresthesia (area under the curve <AUC> = 0.669), feeling of cold feet (AUC = 0.693) and cold skin (AUC = 0.625), or ABI value (AUC = 0.652) in the assessment of clinical status after 6 months of physical training in PAD patients (Figure 3).

## 4. Discussion

PAD can be defined as a cardiovascular continuum, with pathophysiological processes in an ongoing progression from the appearance of atherosclerotic lesions to the onset of acute lower limb ischemia [4]. Referral of patients with vascular artery disease to specialized CR centers is essential to maintain functional status, increase quality of life, and improve long-term prognosis. In our study, we investigated, in a PAD cohort, potential predictors associated with clinical improvement at the 6-month follow-up after starting physical training. The statistical analysis demonstrated that improvement in carbohydrate and lipid profile, of clinical signs, cardiopulmonary testing parameters, or AASI are factors associated with improved clinical status for patients with PAD.

Of the study cohort, 66.7% of the patients were male, with an average age of 69.66 ± 8.60 years (most of the patients are aged in the decades sixth and seventh). These aspects are consistent with existing data in the literature, as it is known that PAD predominantly affects patients over 50 years of age. In terms of sex predominance, recent studies have shown an equal prevalence between males and females [19,20]. In our study, older age, the increased percentage of hypertensive patients (62.9%), and the elevated AASI values (0.52 ± 0.18 at enrollment) were interrelated and mutually reinforcing. The large arteries of elderly hypertensive patients stiffen due to wave reflections, leading to an increase in systolic and pulse pressures [21].

The majority of patients enrolled in the study had a high number of cardiovascular risk factors, which negatively impacts prognosis and increases the risk of an acute cardiovascular event, therefore emphasizing the need to enroll them in special CR programs. Hypertension (*p* = 0.002), diabetes mellitus (*p* = 0.002), dyslipidemia (*p* = 0.045), and obesity (*p* = 0.564) were more frequent in the group of patients without clinical improvement at the 6-month follow-up.

Of the patients, 57.7% had diabetes mellitus, most with inadequate glycemic control (HbA1C 7.96 ± 2.23 g%). Diabetes mellitus is associated with a 2-fold increased risk of developing PAD, coronary artery disease, or ischemic stroke and is associated with an increased risk of arterial stiffness or microcirculation abnormalities [16,22]. Patients with diabetes mellitus have inferior ABI resting values due to the diminished sensitivity [23]. The relationship between diabetes mellitus and arterial stiffness is bidirectional, the latter being considered a risk factor for developing diabetes mellitus [24]. Arterial stiffness precedes an increase of fasting blood glucose [24] and is an independent predictor of all-cause mortality (*p* < 0.001) and cardiovascular-mortality (*p* < 0.001) in patients with type 2 diabetes mellitus [25]. Clinical studies have demonstrated the predictive role of arterial stiffness in the development of CVD in patients with type 2 diabetes mellitus. Several studies have evaluated the potential beneficial role of sodium-glucose co-transporter-2 (SGLT2) inhibitors on the improvement of arterial stiffness. Decreased PWV values have been documented after treatment with dapagliflozin (*p* <0.05) or canagliflozin (*p* < 0.05). Empagliflozin decreased both pulse pressure (compared with baseline) (*p* < 0.001) and AASI (*p* = 0.059) [26].

Umpierre et al. conducted a meta-analysis of randomized controlled clinical trials based on structured exercise training regiments (such as aerobic or resistance) and dietary advice and evaluated the effect of CR programs on serum HbA1C levels in patients with type 2 diabetes mellitus [27]. They demonstrated that exercise sessions of more than 150 min per week were associated with a drop in HbA1C of 0.89% compared with programs lasting less than 150 min, where the drop was significantly lower (0.36%). By comparison with the control group, physical training combined with proper nutrition resulted in a 57.5% decrease in HbA1C [27]. A similar result was obtained in our study, in which we demonstrated that a decrease in HbA1C by at least 4% (*p* = 0.001) and fasting glucose by at least 7% (*p* = 0.001) from baseline is statistically significant for stagnation of functional decline and improving arterial stiffness as a result of improved endothelial dysfunction and decreased pro-inflammatory or pro-oxidative processes.

Arterial stiffness is independently associated with PAD and could be included in the diagnostic and risk stratification strategy in these patients given the increased risk of a major cardiac event [9]. Age, the presence of diabetes, smoking, kidney dysfunction, or lack of exercise are factors associated with arterial stiffness, with Claridge et al. suggesting that it should be considered an early marker of atherosclerosis based on the qualitative and quantitative structural changes of the arterial wall in this category of patients [11,28,29,30].

The analysis of the determinants of arterial stiffness helps risk stratification, therapeutic management, and secondary prevention in PAD, its role as a predictor of mortality and morbidity being recognized in several studies until now [31]. Arterial stiffness is a useful tool in the assessment of organ damage in hypertensive patients. AASI has been introduced as a clinical tool useful in assessing the risk of cardiovascular events. AASI correlates positively with age, systolic BP, and pulse pressure and should be seen as a composite index which indirectly provides details of cardiovascular properties, BP variability, and diurnal cycle [32].

Pulse pressure and AASI are markers of arterial stiffness. In our study, we monitored the blood pressure profile both before the start of the cardiovascular rehabilitation program and at 6 months and found improvement in both pulse pressure (58.65 ± 11.54 mmHg vs. 51.62 ± 12.19 mmHg, *p* = 0.024) and AASI (0.42 ± 0.19 vs. 0.47 ± 0.11, *p* = 0.008). Moreover, with the help of logistic regression, we concluded that the improvement of these two determinants following physical training and multidisciplinary management are associated with clinical improvement and lessening of the arterial stiffening process in PAD patients. The improvement of these parameters is linked to the increased pulsatile flow and enhanced bioavailability of nitric oxide secondary to regular physical training. Vascular compliance decreases with age, leading to increased systolic BP and reduced diastolic BP, making pulse pressure an easily quantifiable clinical marker which reflects decreased vascular compliance [17,33]. The interconnection between arterial stiffness, hypertension, and obstructive sleep apnea has been analyzed in the literature, concluding that approximately 70% of OSA patients are hypertensive and that 25% of them had a brachial pulse pressure over 60 mmHg, indirect marker of arterial stiffness [34]. In hypertensive patients with coronary artery disease (CAD) or kidney dysfunction, arterial stiffness mediates the relationship between pulse pressure and age-adjusted glomerular filtration rate. [35] In our study, the percentage of patients with CAD (59.1% vs. 46.7%) and chronic kidney disease (45.5% vs. 57.3%) was high in both groups, which explains the high pulse pressure values. Vascular calcifications associated with age and pathological entities presented above influence the occurrence and progression of arterial stiffness [36]. Intrinsic stiffness of vascular smooth muscular cells induces the overproduction of collagen and elastin in order to maintain vascular homeostasis [37].

Arterial remodeling is influenced by increased pulse pressure, determining in a secondary level increased wall thickness and the promotion of the atherosclerotic process through the development of plaques [38]. In our study, vascular impairment was frequently associated with PAD, both in patients with clinical improvement or in those without obvious clinical benefit after 6 weeks of exercise training; 68.7% of patients had associated coronary artery disease and 40% had concomitant carotid artery disease. Calcification of coronary arteries is associated with a pro-inflammatory status through activation of several triggers such as smoking or hypertensive peaks [39]. Wilkins et al. demonstrated that low ABI values correlate in a statistically significant way with decreased small artery elasticity in healthy patients [40]. The same relationship was highlighted by Duprez et al. [41] in a study conducted on 43 PAD patients, which concluded that ABI values correlate with small artery elasticity, but not with large artery elasticity. The multidisciplinary approach of these cases is the prerequisite for a favorable clinical and therapeutic outcome, and an integrative approach is necessary in order to avoid polymedication, possible interactions between drugs, or decreased adherence to treatment.

Obesity and dyslipidemia are also associated with a negative prognostic and functional decline leading to a decrease in quality of life and self-independence in PAD [1]. Both adiposity and arterial stiffness are risk factors of CVD; 62.69% of the patients enrolled in our study were obese. The 6-month follow-up showed a statistically significant decrease of the average value of BMI (*p* = 0.037), regular physical training, and proper nutrition, leading to an improved functional status and increased quality of life. The benefits of physical training translate into reduced BMI and body fat, improved blood pressure profile (systolic BP: *p* = 0.034, diastolic BP: *p* = 0.009, pulse pressure: *p* = 0.024, AASI: *p* = 0.008), increased exercise capacity (VO_2_ peak: *p* = 0.034), and improved lipid and carbohydrate parameters (LDL-cholesterol: *p* = 0.004, triglycerides: *p* = 0.005, fasting glucose: *p* < 0.001, HbA1C: *p* < 0.001). Between these parameters there is a reciprocal potentiation effect, also evident in our study, which is explainable by the multiple implications of endothelial dysfunction, the implication of the of the renin-angiotensin-aldosterone system as well as inflammatory processes, which determine both intrinsic changes in the quality of the arterial wall and its thickness. Strasser et al. demonstrated that abdominal obesity and visceral fat are positively associated with large artery stiffness [29]. The accumulation of abdominal fat in adulthood causes endothelial dysfunction and its distribution influences the process of arterial stiffening. Android fat is correlated with arterial stiffness but gynoid fat is not. This pattern suggests that arterial stiffness is independent of total fat mass and that there are differences in terms of morphology or pathophysiological mechanisms vascular mediators such as adipocyte size, adipokines, pro-inflammatory cytokines, or lipid species that may cause arterial stiffening [42,43]. Free fatty acids, insulin, leptin, and pro-inflammatory cytokines are circulating factors with high serum levels in obese patients that cause, through the stimulation of vascular smooth muscle proliferation, endothelial dysfunction and increased sympathetic activity and indirectly the progression of the arterial stiffening process [44].

Montero et al. conducted a meta-analysis on the effect of CR programs on arterial stiffness in obese patients (with a BMI over 30 kg/m^2^), enrolling a total of 235 patients from eight trials. Arterial stiffness parameters were assessed 8 weeks after the start of physical training, concluding that aerobic training does not induce a statistically significant reduction in them (*p* = 0.14). The reduction of the arterial stiffening process was observed in association with the decrease of SBP in a subgroup of obese patients who underwent a low-intensity aerobic training (*p* < 0.01), but further research is needed in the area [45,46]. Marcell et al. [47] emphasizes the idea of a beneficial effect of physical training on the process of arterial stiffening by lowering serum levels of pro-inflammatory factors only in the case of weight loss in obese patients. In our study, we observed a statistically significant reduction in inflammatory markers (CRP: *p* = 0.047) and BMI (*p* = 0.037) in PAD patients with clinical improvement at the 6-month follow-up, which supports the data from the literature.

Smoking is the main risk factor associated with PAD and through systemic and vascular inflammation, endothelial function, and damage to the extracellular matrix affect arterial elastic properties, which influence the arterial stiffness [48,49]. In our study, 33.3% of the patients were smokers at the time of inclusion, and 34% admitted to previous smoking. The logistic regression concluded that smoking cessation has a beneficial effect on both functional status and quality of life, being an independent predictor at 6 months associated with clinical improvement in patients with PAD (*p* < 0.001). Smoking cessation leads to improvement of pulse pressure and AASI by relieving endothelial dysfunction and antagonizing pro-inflammatory status and increased tissue absorption of smoke particles [49]. Armstrong et al. demonstrated in a cohort of 739 PAD patients who underwent lower limb angiography that continuing smoking despite interventional treatment was associated with an increase in the 5-year mortality rate (31% vs. 14%, *p* < 0.05%) [50]. In another study, Fu et al. proved the independent relationship between smoking and peripheral artery stiffness in PAD asymptomatic patients [51]. In a systematic review including 39 clinical trials, Doonan et al. [52] concluded that active smoking leads to a significant increase in the degree of arterial stiffness. Chronic smoking is a risk factor associated with increased arterial stiffness [53], but to date clinical trials have not shown statistically significant differences between smokers and non-smokers. Smoking cessation leads to a slowing of the arterial stiffening process, but the long-term effect requires further studies [54].

In our study, the biological assessment carried out at enrolment revealed elevated serum levels of lipid profile (both total cholesterol, LDL-cholesterol, HDL-cholesterol, and serum triglycerides), fasting blood glucose, and inflammatory markers (CRP). The improvement in mean serum values of these parameters secondary to the CR program suggests the beneficial role of physical training associated with multidisciplinary management. Lipid-lowering medication and drug treatment of diabetes mellitus improve the prognosis of patients with PAD, leading to decreased mortality rate or cardiac hospitalizations [55,56]. A similar role is played by antihypertensive treatment, as maintaining blood pressure values within normal limits has pathophysiological correspondences in the progression and development of atherosclerosis-related complications. Several studies reported that high triglyceride/HDL-cholesterol ratio positively correlates with arterial stiffness. Special attention is paid to serum triglyceride levels, which represent a risk factor for arterial stiffness independent of the cardiovascular risk and liver function [57]. In our study, we demonstrated that decreasing triglycerides by at least 13% (*p =* 0.026) results in improvement of clinical parameters and of parameters associated with arterial stiffness (AASI and pulse pressure).

In our cohort, we demonstrated that decreasing serum levels of LDL-cholesterol by at least 10% (*p* = 0.013) and triglycerides by at least 13% (*p* = 0.026) are favorable predictors for clinical improvement at the 6-month follow-up. Studies have demonstrated that inflammatory markers such as C-reactive protein, tumor necrosis factor, or interleukin 6 have a statistically significant correlation with arterial stiffness [58]. In our cohort, although the decrease of CRP serum levels was significant in the group of patients with clinical improvement (2.87 ± 2.77 mg/dL vs. 5.30 ± 9.26 mg/dL, *p* = 0.047), the statistical analysis did not reveal this parameter as an independent predictor (*p* = 0.086).

The referral of PAD patients to specialized cardiovascular rehabilitation centers is low, increasing adherence to treatment, awareness of the importance of enrollment in such programs, and the beneficial results both physical and psychological being the goals of the work presented. The benefits of physical training translated into increased walking distance (*p* = 0.046) and ABI values (*p* = 0.031) at the 6-month follow-up.

In PAD, arterial stiffness is associated with walking distance in treadmill test [59]. In our study, we evaluated the AASI both before and at the 6-month follow-up after starting physical training. Patients who showed clinical improvement associated higher ABI values (*p* = 0.031) and lower AASI values (*p* = 0.008), results similar to those presented in the literature. Brewer et al. demonstrated that increased brachial pulse pressure and higher aortic augmentation index are associated with functional decline and implicitly with decreased quality of life by limiting daily activities [60]. Arterial stiffness defined by an increased brachial-ankle PWV predicts walking distance after adjustment for ABI value or cardiovascular risk factors [61]. Vascular stiffness has hemodynamic consequences that limits exercise capacity [61]. Several studies have so far demonstrated that improving endothelial dysfunction, combating pro-inflammatory and pro-thrombotic status as well as decreasing sympathetic tone combat arterial stiffness and improve the results of physical training [62,63]. Tanaka et al. concluded that regular physical training, low salt intake, weight loss, and limiting alcohol consumption are factors that contribute to reducing arterial stiffness in PAD [64].

The benefits of physical training are noticeable on a morphological level, a hemodynamic one, as well as on a metabolic level. The main objective of our study was to assess exercise capacity (expressed as VO_2_ peak). Cardiopulmonary testing performed 6 months after the start of the CR program showed a significant increase in VO_2_ peak in patients of the first group (14.95 ± 5.74 mL/kg/min vs. 12.13 ± 3.94 mL/kg/min, *p* = 0.034). The increasing of VO_2_ peak occurs secondary to increased cardiac output (as central mechanism) and O_2_ extraction (as peripheral mechanism). Regular exercise decreases sympathetic activity, enhances the parasympathetic nervous system, and modulates arterial baroreflex function, leading to lower blood pressure at exercise and rest and decreased heart rate variability. In our study, at the 6-month assessment we observed a reduction in peak systolic blood pressure at cardiopulmonary testing (154.94 ± 18.11 mmHg, 164.76 ± 23.55 mmHg, *p* = 0.040), this being an independent predictor associated with clinical improvement 6 months after the start of a recovery program (OR 0.974, 95% CI 0.949–0.999, *p* = 0.045). At the peripheral vascular level, regular exercise improves NO-mediated vasodilation and stimulates endothelial progenitor cell formation, with increased arteriolar diameter and improved O_2_ extraction. These changes are clinically translated into improved Borg Scale score as we demonstrated in our study using logistic regression (*p* = 0.004). In addition to increasing exercise capacity, physical training is associated with a decrease in arterial stiffening due to improvements in nitric oxide bioavailability, which causes an increased vasodilatory effect. These issues underpin the improvement of both AASI and the VO_2_ peak, thus contributing to the concept of arterial “de-stiffening” and improving endothelial function [65].

Our study has several limitations due to the relatively small number of cases analyzed and variability in the assessment of improvement of clinical signs. We excluded those records where critical information was unavailable. This was done to minimize the risk of misclassification, introducing a limited risk of selection bias. Further on, the clinical improvement may be attributable to other various factors besides those that were mentioned in our study. As well, the 6-month limited time monitorization data may be modified on a longer follow-up.

## 5. Conclusions

In our study we demonstrated that improving carbohydrate and lipid profile, adopting a healthy lifestyle, quitting smoking, and improving clinical signs as well as increasing exercise capacity were central elements associated with preserving functional status, decreasing the arterial stiffening process, and increasing quality of life in patients with PAD that underwent CR programs. Based on the data presented, the design of an integrative algorithm to evaluate the effectiveness of CR programs may represent a future research direction.

## Figures and Tables

**Table 1:** Demographics and resting hemodynamics.

Parameter	Initial Evaluation (*n* = 97)	6-Month Follow-Up (*n* = 97)	*p* Value
**Anthropometric Parameters**			
Height, cm	171.44 ± 8.77	-	
Weight, kg	83.72 ± 15.49	73.08 ± 13.52	0.708
Body mass index, kg/m^2^	27.74 ± 3.65	24.14 ± 3.17	0.037
**Resting hemodynamics**			
Heart rate, bpm	72.71 ± 12.28	68.55 ± 11.98	0.096
Systolic BP, mmHg	140.79 ± 21.56	134.73 ± 20.63	0.131
Diastolic BP, mmHg	80.15 ± 14.97	77.26 ± 14.43	0.485
**Sedentary lifestyle**	69 (71.1%)	51 (52.6%)	0.032
**Smoking**			
Active or in the past	63 (64.9%)	29 (29.9%)	<0.001
**Alcohol**	16 (16.5%)	9 (9.3%)	0.973

All values are expressed as mean ± standard deviation (SD) or *n* (%); bpm: beats per minute; BP: blood pressure.

**Table 2:** Comorbidities and clinical examination.

Parameter	6-Month Follow-Up (*n* = 97)	No Clinical Improvement after CR Program (*n* = 22)	With Clinical Improvement after CR (*n* = 75)	*p* Value
**Comorbidities**				
Hypertension	61 (62.9%)	20 (90.9%)	41 (54.7%)	0.002
Diabetes mellitus	56 (57.7%)	19 (86.4%)	37 (49.3%)	0.002
Dyslipidemia	57 (58.8%)	17 (77.3%)	40 (53.3%)	0.045
Obesity	61 (62.9%)	15 (68.2%)	46 (61.3%)	0.564
Coronary artery disease	48 (49.5%)	13 (59.1%)	35 (46.7%)	0.310
Heart failure	70 (72.2%)	18 (81.8%)	52 (69.3%)	0.255
COPD	14 (14.4%)	6 (27.3%)	8 (10.7%)	0.052
Chronic kidney disease	53 (54.6%)	10 (45.5%)	43 (57.3%)	0.330
**Clinical picture—improvement of signs and symptoms**				
Paresthesia	43 (44.3%)	4 (18.2%)	39 (52.0%)	0.005
Feeling of cold feet	51 (52.6%)	5 (22.7%)	46 (61.3%)	0.001
Pale skin	35 (36.1%)	9 (40.9%)	26 (34.7%)	0.596
Cold skin	32 (33.0%)	3 (13.6%)	29 (38.7%)	0.028
Reduced pilosity	32 (33.0%)	4 (18.2%)	28 (37.3%)	0.254
Subcutaneous atrophy	21 (21.6%)	2 (9.1%)	19 (25.3%)	0.106
Thickened nails	22 (22.7%)	3 (13.6%)	19 (25.3%)	0.254
Petechiae	21 (21.6%)	4 (18.2%)	17 (22.7%)	0.657
Arterial ulcers	5 (5.2%)	1 (4.5%)	4 (5.3%)	0.885
Dermatitis	12 (12.4%)	3 (13.6%)	9 (12.0%)	0.840
**Quitting smoking**	29 (29.9%)	15 (68.2%)	61 (81.3%)	<0.001
**Active lifestyle**	51 (52.6%)	16 (72.7%)	35 (46.7%)	0.032

All values are expressed as n (%). COPD: chronic obstructive pulmonary disease.

**Table 3:** Blood biochemistry and exercise stress test parameters.

Parameter	Before CR	6-Month Follow-Up (*n* = 97)	With Clinical Improvement after CR (*n* = 75)	No Clinical Improvement after CR Program (*n* = 22)	*p* Value
**Blood biochemistry**					
Total cholesterol, mg/dL	206.95 ± 75.90	172.92 ± 63.12	167.95 ± 58.19	189.88 ± 76.76	0.153
LDL-cholesterol, mg/dL	135.40 ± 67.79	121.86 ± 61.01	112.43 ± 43.58	154.02 ± 94.47	0.004
HDL-cholesterol, mg/dL	42.84 ± 16.92	44.56 ± 17.60	44.08 ± 18.66	46.18 ± 13.63	0.625
Triglycerides, mg/dL	185.27 ± 122.13	161.18 ± 106.26	145.10 ± 61.93	216.01 ± 184.46	0.005
CRP, mg/dL	5.33 ± 14.30	3.42 ± 5.07	2.87 ± 2.77	5.30 ± 9.26	0.047
HbA1C, g%	7.96 ± 2.23	7.64 ± 2.14	6.71 ± 1.35	10.75 ± 1.16	<0.001
Fasting glucose, mg/dL	153.94 ± 63.54	146.51 ± 64.19	132.65 ± 43.85	193.13 ± 94.89	<0.001
**Exercise stress test**					
VO_2_ peak mL/kg/min	12.34 ± 4.74	14.31 ± 4.74	14.95 ± 5.74	12.13 ± 3.94	0.034
Peak HR, bpm	118.61 ± 18.81	131.66 ± 20.88	132.66 ± 21.54	128.25 ± 18.48	0.386
Peak systolic BP, mmHg	171.52 ± 27.03	157.17 ± 19.78	154.94 ± 18.11	164.76 ± 23.55	0.040
Peak diastolic BP, mmHg	90.54 ± 13.31	88.73 ± 13.04	89.40 ± 13.46	86.46 ± 11.50	0.356
RER	1.07 ± 0.10	1.09 ± 0.10	1.076 ± 0.10	1.070 ± 0.10	0.820
Borg scale	15.45 ± 1.58	13.31 ± 1.14	13.12 ± 1.02	13.95 ± 1.29	0.002
**ABI**	0.65 ± 0.21	0.66 ± 0.23	0.84 ± 0.25	0.71 ± 0.24	0.031
**Walking distance, m**	246.43 ± 185.49	274.96 ± 175.52	291.33 ± 174.87	219.15 ± 169.84	0.046
**Echocardiography**					
LVEF, %	46.41 ± 9.74	49.37 ± 10.43	50.93 ± 9.93	44.03 ± 10.55	0.006
**Blood pressure monitoring, 24 h**					
Systolic BP	143.60 ± 21.99	135.42 ± 20.74	129.67 ± 15.52	138.27 ± 19.58	0.034
Diastolic BP	81.59 ± 15.24	77.59 ± 14.50	71.40 ± 11.32	79.28 ± 15.17	0.009
Pulse pressure	81.59 ± 15.24	77.59 ± 14.50	58.65 ± 11.54	51.62 ± 12.19	0.024
AASI	0.52 ± 0.18	0.46 ± 0.12	0.42 ± 0.19	0.47 ± 0.11	0.008

All values are expressed as mean ± standard deviation (SD). LDL-cholesterol: low-density lipoprotein cholesterol, HDL-cholesterol: high-density lipoprotein cholesterol; CRP: C reactive protein; HbA1C: glycated hemoglobin; VO_2_ peak: peak oxygen uptake; bpm: beats per minute; HR: heart rate; BP: blood pressure; RER: respiratory exchange ratio; ABI: ankle-brachial index; LVEF: left ventricle ejection fraction; AASI: ambulatory arterial stiffness index.

**Table 4:** Predictors of clinical improvement at the 6-month follow-up.

	Logistic Regression		
Parameter	OR	*p* Value	95% CI
Decreasing fasting glucose by at least 7%	0.986	0.001	0.978–0.995
Decreasing of HbA1C by at least 4%	1.097	0.001	1.039–1.159
Changing sedentary lifestyle	0.328	0.036	0.116–0.930
Ceasing smoking	0.107	<0.001	0.037–0.312
Improvement of paresthesia	4.875	0.008	1.507–15.775
Improvement of feeling of cold feet	5.393	0.003	1.795–16.204
Improvement of cold skin	3.993	0.037	1.085–14.699
Decreasing LDL–cholesterol by at least 10%	0.989	0.013	0.981–0.998
Decreasing triglycerides by at least 13%	0.994	0.026	0.988–0.999
Increasing of ABI value	10.753	0.034	1.196–96.685
Increasing Borg Scale score	0.446	0.004	0.259–0.769
Increasing of VO_2peak_ mL/kg/min	1.138	0.040	1.006–1.287
Decreasing of peak systolic BP, mmHg	0.974	0.045	0.949–0.999
Improvement of pulse pressure	0.954	0.028	0.915–0.995
Improvement of AASI	1.127	0.021	1.069–1.105

CI: Confidence interval; OR: Odds ratio; HbA1C: glycated hemoglobin; LDL-cholesterol: low-density lipoprotein cholesterol; ABI: ankle-brachial index; VO_2_ peak: peak oxygen uptake; BP: blood pressure; AASI: ambulatory arterial stiffness index.
